# A Case of Adrenal Metastasis in Seminoma

**DOI:** 10.1155/2013/495743

**Published:** 2013-08-21

**Authors:** Bouabdallah Zakaria, Théveniaud Pierre-Etienne, Duchatelle Véronique, Molinié Vincent, Massoud Walid, Baumert Hervé

**Affiliations:** ^1^Department of Urology, Paris Saint Joseph Hospital Group, 185 Rue Raymond Losserand, 75014 Paris, France; ^2^Department of Pathology, Paris Saint Joseph Hospital Group, 185 Rue Raymond Losserand, 75014 Paris, France

## Abstract

We report an uncommon case of testicular cancer with adrenal metastasis without retroperitoneal or distant metastatic disease. This situation is highly unusual. In fact, no similar case was reported in the literature. Our case is the first adrenalectomy that has been performed for secondary localization of testicular cancer. After eighteen-month followup, the patient was doing well, with no evidence of disease.

## 1. Case 

**Figure 1:**
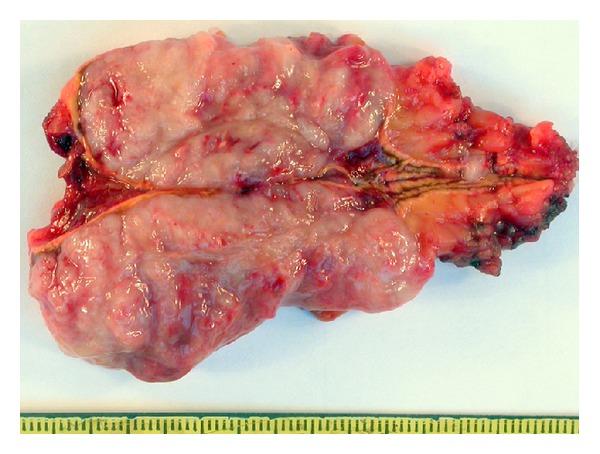
Macroscopic aspect of the tumor.

**Figure 2:**
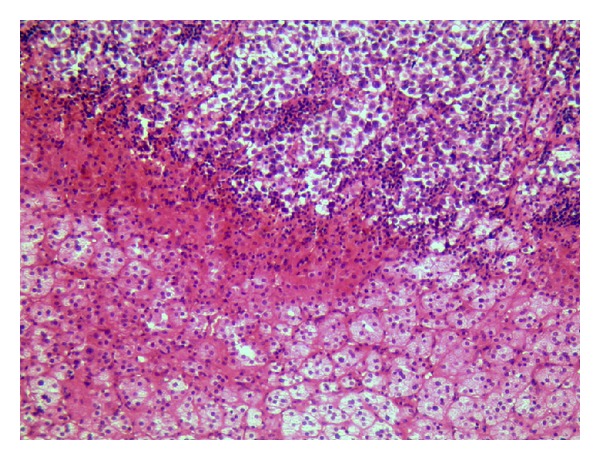
Standard coloration with hematoxylin and eosin showing both tumor and normal adrenal glands (×20).

**Figure 3:**
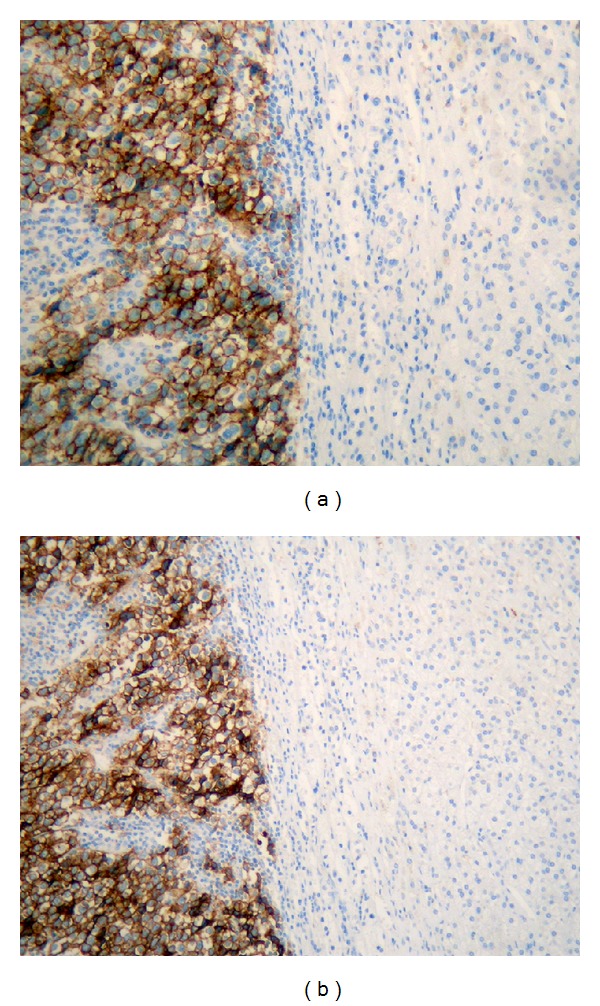
Seminoma cells markers expression (a) CD 117 and (b) PLAP.

A 53-year-old patient underwent a right radical orchiectomy after he noted a testicular mass on self-examination. Pathology revealed a testicular seminoma. He received 3 cycles of bleomycin, etoposide, and cisplatin associated with radiotherapy of iliac and retroperitoneal lymph nodes. Five years later, he presented a contralateral seminoma, which was treated by radical orchiectomy and 4 cycles of etoposide and cisplatin. A CT scan Nine years later showed a mass of the right adrenal gland with fixation at PET scan associated with a preaortic lymph node fixation. Tumor markers were negative (alpha-fetoprotein and beta-human chorionic gonadotrophin). No evidence was found for other distant metastases. A right adrenalectomy with lymph node dissection was performed by laparoscopy. Pathologic examination showed an adrenal metastasis of a testicular seminoma without lymph node metastasis (Figures 1, 2, and 3). The patient is disease free at last followup 18 months after surgery.

## 2. Comment

Testicular cancer is the first solid tumor in young men between 20 and 30 years old [1]. Seminoma has a high metastatic capacity. Metastases initially occur in the retroperitoneal nodes before additional systemic involvement [2]. The most common metastatic sites of germinal tumors are the lungs, liver, central nervous system, and bone [2, 3]. Other localizations are atypical. Cases of isolated metastases were reported in the literature: spleen, skin, prostate [4–6], and so forth. Isolated adrenal metastasis of seminoma is uncommon, and, to our knowledge, this is the first case reported. PET scan can help diagnose difficult cases [7]. The treatment of metastasis is based on chemotherapy. However, surgery may be indicated in the following cases: diagnostic doubt, functional repercussion, or residual mass [3].
